# Exploring nudging strategies for plant-based dietary choices in hospital patients: a quasi-experimental study

**DOI:** 10.1186/s12966-025-01793-w

**Published:** 2025-07-01

**Authors:** Kristin Hünninghaus, Hannah Caroline Schäfer, Maik Plonka, Rebeca Montejano Vallejo, Gustav Dobos, Heidemarie Haller

**Affiliations:** Center for Integrative Medicine and Planetary Health, University Hospital Essen, University of Duisburg-Essen, Essen, Germany

**Keywords:** Nudging, Planetary health diet, Dietary choice, Hospital catering, Sustainability, Sustainable eating

## Abstract

**Background:**

The global food system is responsible for climate change, biodiversity loss, and land-use changes. At the same time, the prevalence of diet-related, chronic diseases is increasing worldwide. A dietary shift to a plant-based diet could protect both planetary and individual health. Nudging can positively influence dietary choices. We investigated how different nudges influenced inpatients’ choices of plant-based menus.

**Methods:**

A quasi-experimental study was conducted across three consecutive four-week phases at the University Hospital Essen, Germany. In the baseline phase, inpatients chose meals from a standard menu without any intervention. In a second phase, an order nudge was applied by listing the plant-based dish first on the menu. In the third phase, a combined nudge was applied, adding a verbal recommendation to the order nudge. Data from 6,575 inpatients (mean age: 57.3 ± 18.7; 50.6% female) covering 26,949 meal choices were analyzed using logistic regression and generalized linear modeling.

**Results:**

Independent of nudging, female sex and younger age predicted plant-based menu choices (*p* <. 001, respectively). Controlling for the effects of sex and age, patients were nearly twice as likely to choose the plant-based menu during both intervention phases compared to baseline (order nudge: OR = 1.95; 95% CI [1.55–2.45]; *p* <.001; combined nudge: OR = 1.95; 95% CI, [1.56–2.44]; *p* <.001). However, there was no significant difference in plant-based menu selection between the two nudges (OR = 1.00; 95% CI [0.80–1.25]; *p* =.992). Subgroup analyses further revealed that both women and men as well as middle-aged (36–64 years) and older adults (≥ 65 years), but not younger adults (18–35), were significantly more likely to select plant-based menus during the interventions compared to baseline. Among both sexes and across all age groups, no differences in plant-based meal selection were found between the order and the combined nudge (all *p* >.001).

**Conclusion:**

Centrally implemented nudging is a simple and effective strategy that can increase patients’ choice of plant-based menus, which in turn may promote patient health and contribute to positive environmental outcomes.

**Trial registration:**

DRKS00036763. Registered 29 April 2025.

**Supplementary Information:**

The online version contains supplementary material available at 10.1186/s12966-025-01793-w.

## Background

The global food system is largely responsible for the climate and biodiversity crisis as well as for diet related chronic diseases. Shifting to a plant-based diet aligning with the Planetary Health Diet (PHD) can protect both planetary and individual health. The PHD, advocated by the EAT-Lancet commission, promotes a diet heavy in fruits, vegetables, whole grains, nuts and legumes, with relatively low intakes of animal-derived foods such as meat and dairy [1]. While plant-based dietary habits have long been part of traditional eating habits– such as the Mediterranean diet– a novel aspect of the PHD is its explicit integration of nutrition within global boundaries and environmental sustainability goals [2].

Currently, food production accounts for around 30% of total emissions, with 83% of those emissions in the EU stemming from meat, egg and dairy production [3, 4]. Shifting to more plant-based dietary habits could significantly reduce greenhouse gas emissions [2]. In addition, following a plant-based diet is associated with a lower risk of obesity and diet-related secondary diseases such as type 2 diabetes mellitus and cardiovascular disease [5, 6, 7, 8]. While 14% of all deaths in Germany are associated with an unhealthy diet, switching to a plant-based diet could prevent around 150,000 deaths annually [9, 10].

Despite the benefits associated with the PHD, research indicates suboptimal adherence. A Brazilian study, for example, found that the population remains far from achieving the recommended intake levels [11]. In France and the Netherlands, even participants with the highest adherence scores to PHD-based indices showed insufficient intake of key plant-based foods and nutrients, indicating that current diets fall short of PHD targets [12, 13]. Overall, PHD adherence seems to be low across Northern and Central Europe [14]. Specifically, in 2019, one-third of the EU population (33%) did not consume any fruit or vegetables daily, which are fundamental components of the PHD. Only 12% met the recommended intake of five or more portions per day [15]. In Germany, 45.1% of women reported eating fruit and vegetables daily, compared to only 24.1% of men [16]. In general, available data suggest that women and younger individuals are more likely to adopt plant-based dietary patterns [17, 18, 19, 20]. However, the findings underscore both an existing gap and the potential for improvement in aligning dietary habits with the PHD.

In today’s Western world, where food is available 24/7, the responsibility for a healthy diet cannot rest solely on the individual. Creating a healthy food environment therefore requires significant public and political action [21]. Hospitals have a profound responsibility here: in addition to providing direct medical care, they must create an environment that promotes and supports a healthy diet as part of a broader approach to public health, prevention, and education. Moreover, hospitals must also address their substantial ecological footprint. While their emissions account for up to 29% of the health sector’s carbon footprint [22], a life cycle assessment of 33 acute hospitals in Switzerland revealed that, for the average hospital, catering was responsible for 17% of its climate impact [23].

Nudging towards more plant-based food choices in hospital settings could contribute to the health promotion of patients and at the same time to greater sustainability in the healthcare sector. Nudging is defined as “any aspect of the choice architecture that alters people’s behavior in a predictable way without forbidding any options or significantly changing their economic incentives” (Thaler & Sunstein, 2009, p.6, as cited in [24]). Healthy eating nudges comprise easy, affordable and freedom-preserving changes to the environment, such as increasing the visibility of healthy food [25, 26], reorganizing menu plans to place the healthier option first [27, 28, 29, 30, 31], and promoting the plant-based option as the chef’s or restaurant’s recommendation of the day [32, 33].

Across both physical and digital settings, placing vegetarian or plant-based options first have shown to significantly increase their selection. Andersson and Nelander (2021) [27] and Dayan and Bar-Hillel (2011) [28] found that menu order alone led to reductions in meat choices and increased selection rates for early-listed, healthy items. When placing healthy items at different lateral positions, Romero et al. (2026) found that consumers were more likely to choose healthy items when displayed to the left vs. right of the unhealthy food item [31]. More recent work by Wongprawmas et al. (2023) [30] and Franchini et al. (2023) [29] confirms these effects in institutional and online environments, suggesting that menu placement acts as a powerful, low-effort nudge to guide selections towards targeted options in different settings. Complementing positional nudges, labeling vegetarian dishes as the “Chef’s Recommendation” increased the odds of infrequent vegetarian eaters choosing those dishes [32]. While Bacon & Krpan (2018) [32] had only tested the chef’s recommendation combined with a salience nudge, Weijers et al. (2024) [33] found that chef recommendations alone were effective across different vegetarian meals: participants who received the recommendation nudge chose vegetarian options 1.42 times more often.

Despite growing evidence for the effectiveness of these nudges in public and commercial food environments, it remains unclear how well they translate to clinical settings like hospitals. To date, only one study conducted on a cardiology ward showed that a multicomponent nudging strategy with visual cues and informational nudges (traffic light labels, posters) significantly increased the selection of vegetarian menus as well as side and fruit salads among patients [34]. However, little is known about which specific nudges are most effective, and whether single-component strategies could achieve similar outcomes. Understanding the relative contribution of individual nudges is important for designing low-effort, scalable interventions in clinical settings, where resources such as time and staff are often limited [35, 36].

The objective of this study was to expand the current evidence and explore how nudging strategies in favor of plant-based menus may improve the dietary choices of a hospital patient population, benefiting both individual health outcomes and the environmental impact of hospital food services. Building on previous research highlighting the potential of both menu placement and chef recommendation nudges as simple yet effective strategies to promote plant-based choices across diverse food service settings [27, 28, 29, 30, 31, 32, 33], we first aimed to investigate whether a simple, single-component order-nudge– placing the plant-based menu option first– could influence plant-based dietary choices of inpatients. Given that combined nudges may have stronger effects [37], we then tested whether adding a “chef’s recommendation” nudge would further enhance the selection of plant-based meals among inpatients.

## Materials and methods

### Design

A quasi-experimental study was conducted at the University Hospital in Essen, Germany, from November 2022 to March 2023. A nudging intervention was implemented to encourage hospital inpatients to choose the plant-based dish from the lunch menu. Patients could choose between three dishes: two animal-based options, and one plant-based. The plant-based option thereby differed between being vegetarian or vegan. While printed menus were available at each patient’s bedside to assist with the selection of the lunch menu, a menu assistant visited the patient on a daily basis to present the menu once more orally and to take the order in digital form. Anonymized data were obtained by the digital menu ordering system Ariadne (SE-Software Engineering GmbH). Therefore, informed consent was not required. The study was registered online retrospectively at the German Clinical Trial Register (DRKS00036763).

### Eligibility criteria

The minimum age for participation was set at 18 years. Given the quasi-experimental design of the study, inpatients of different wards with all dietary preferences were included (e.g., omnivore, vegetarian or vegan diet).

### Study intervention

The experiment comprised three phases, each with three respective cohorts over a period of 4 weeks (Table 1). Phase 1 served as the baseline with no nudges applied. During this phase, the animal-based dish was listed first on the printed lunch menu, followed by the second animal-based dish, while the plant-based dish was listed last. In phase 2, an order nudge was introduced: the plant-based dish was listed first on the paper menu plan and was also first to be offered verbally by the menu planners. In phase 3, the plant-based dish was still listed and presented first both on the paper menu as well as by the menu assistant. In addition, an oral recommendation was added: all menu assistants were trained to recommend the plant-based option as being the kitchen’s recommendation of the day. In each phase, the same menus were offered, i.e., the entire 4-week menu for cohort 1 was again offered for cohort 2 and 3, respectively.

**Table 1:** Intervention details

	Type of intervention	Menu Order
Phase 1	Baseline, no nudges applied	Animal-based dish → Light animal-based dish → Plant-based dish
Phase 2	Order nudge	Plant-based dish → Animal-based dish → Light animal-based dish
Phase 3	Order + oral recommendation nudge	Plant-based dish (+ chef’s recommendation) → Animal-based dish → Light animal-based dish

### Outcome measures

The primary outcome was the number of selected menus of each type. Additionally, anonymized demographic data (age, sex, duration of days within a study phase, and the type of wards where inpatients were treated) were recorded. All data were recorded using the hospital’s electronic food ordering system Ariadne.

### Data analysis

The data were exported from the digital food ordering system Ariadne and analyzed using SAS Software version 9.4 and R version 4.4.2 (2024-10-31 ucrt). Because of their different nutritional needs in comparison to somatically ill patients in routine care, inpatients from psychiatric, intensive care and neurosurgical wards as well as those from intermediate care were excluded. We also excluded inpatients who were in more than one intervention phase.

Demographic data were displayed as means and standard deviations. Logistic regression modelling was first conducted to determine whether sex, age or duration of days within a study phase independently influenced plant-based menu choices, regardless of the nudge intervention. Generalized linear modeling accounting for repeated measures within patients was then used to compare menu choices between the three phases. The model included fixed effects for intervention phase, sex and age. A random intercept for patients was added to control for individual differences in baseline plant-based choice probabilities. We specified a binomial distribution with a logit link function to model the binary outcome of plant-basedversus non-plant-based choice.

To run the model, the three menu options were dichotomized: menu one and two (both being animal-based) were combined into a single animal-based category, allowing us to contrast the choices of plant-based and non-plant-based options. Covariates that emerged as potentially significant predictors in the preliminary logistic regression analysis were included in the model to adjust for potential confounding effects. In addition, generalized linear modeling for repeated measures within patients (accounting for age and sex, respectively) was used to conduct subgroup analyses and to examine whether menu choice differed by sex and age within the different phases. Patients’ age was categorized into age groups of young adults (18–35), middle-aged (36–64) and older adults (65 and above). Due to the large sample size, a p-value of < 0.001 was considered significant for all analyses. To explore whether the observed effects in the main sample were also present among patients who were part of more than one phase, we conducted further subgroup analyses.

## Results

### Data selection

**Fig. 1:**
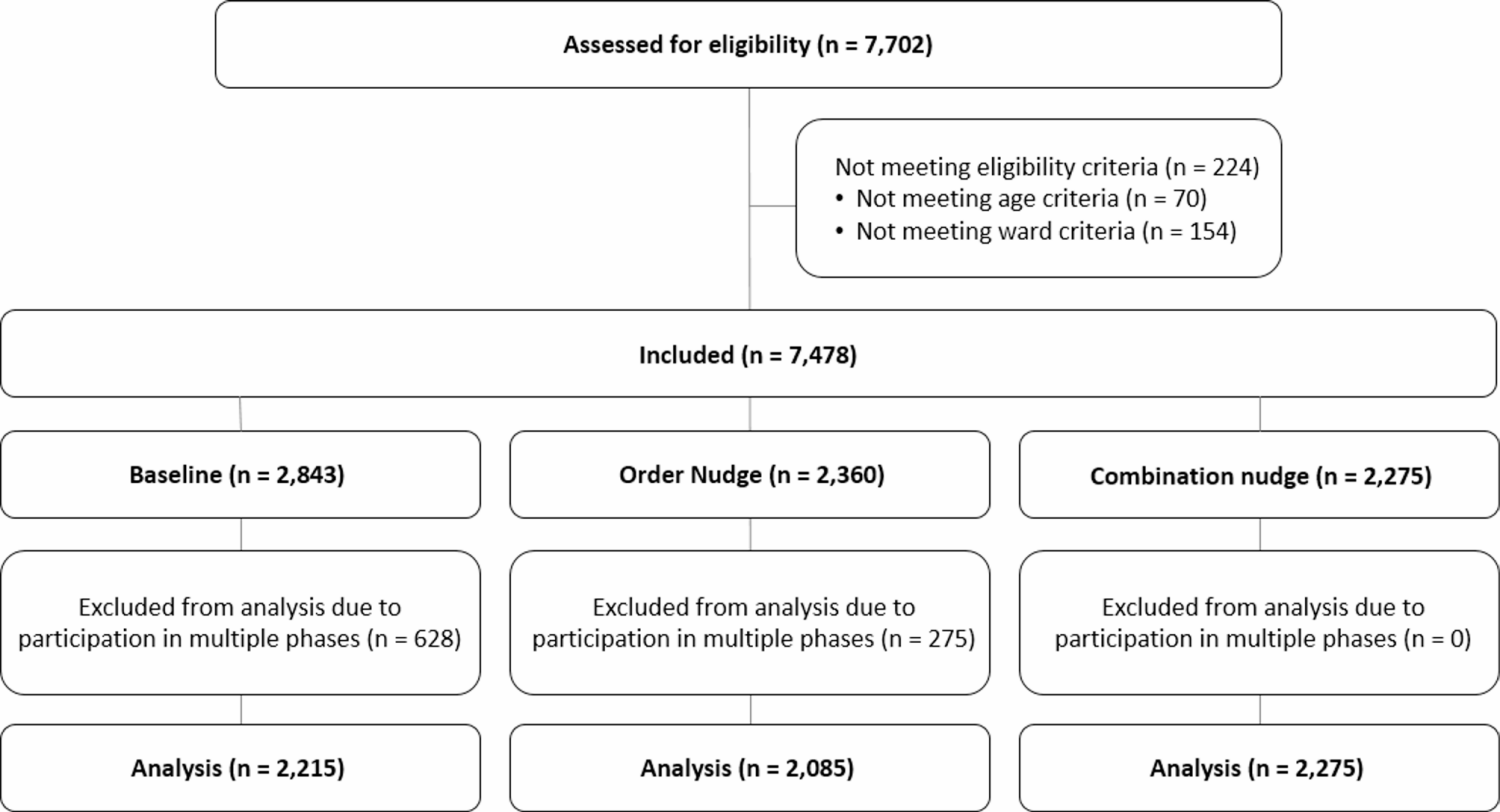
Flow chart of patient inclusion

Of the 41,782 registered menu choices from *n* = 7,702 inpatients, data from patients under 18 years, data from patients who were unable to choose between main menus (e.g., special diets) and data from patients at other than the defined wards were excluded (*n* = 224). Patients who participated in more than one intervention phase were also excluded (*n* = 903). This resulted in a final dataset of 26,949 menu choices from 6,575 patients (Fig. 1).

### Sample characteristics

Table 2 displays the sample characteristics at baseline and across nudging phases. The average age of the 6,575 patients was 57.3 ± 18.7 years. Due to technical errors, the sex data of 17.3% of patients was missing. Of those identified, 50.7% were female, and 49.3% were male. On average, a patient remained in an intervention phase for 4.1 ± 4.1 days.

**Table 2:** General characteristics of the sample by phase of the intervention

	Total (*N* = 6,575)	Baseline (Phase 1) (*N* = 2,215)	Order nudge (Phase 2) (*N* = 2,085)	Order + oral recommendation nudge (Phase 3) (*N* = 2,275)
Age (mean ± SD)	57.30 ± 18.67	57.44 ± 18.67	56.52 ± 18.80	57.87 ± 18.53
Sex: female / male (N)*	2,755 / 2,680	905 / 928	885 / 828	965 / 924
Days in a phase (mean ± SD)	4.10 ± 4.10	4.09 ± 4.12	3.93 ± 3.88	4.25 ± 4.25

* For variable sex, data for 1,140 (17.3%) participants were missing

SD: Standard deviation; N: Number of patients

### Regression analysis of confounding variables

In each phase of the intervention, sex and age were significant predictors of menu choice, with females and younger patients being more likely to choose a plant-based menu (Table 3). The number of days in a phase was not shown to be a significant predictor of the study outcome.

**Table 3:** Logistic regression modeling of confounding variables on plant-based menu choice

	Baseline (Phase 1) (*N* = 2,215)	P*	Order nudge (Phase 2) (*N* = 2,085)	P*	Order + oral recommendation nudge (Phase 3) (*N* = 2,275)	P*
Sex (AOR (95% CI))	0.55 (0.48–0.62)	< 0.001	0.65 (0.57–0.73)	< 0.001	0.70 (0.62–0.78)	< 0.001
Age (AOR (95% CI))	0.98 (0.98 − 0.99)	< 0.001	0.99 (0.98–0.99)	< 0.001	0.99 (0.99–0.99)	< 0.001
Days in a phase (AOR (95% CI))	0.99 (0.98–1.00)	0.121	0.99 (0.98–1.00)	0.105	0.98 (0.99–1.01)	0.619

* P-values were calculated by logistic regression modeling

AOR: Adjusted Odds Ratio; CI: Confidence Interval; N: Number of patients in a phase

### Main effects of nudging

Table 4 displays the numbers of chosen menus by phase of the intervention. Generalized linear mixed models corrected for the influence of sex and age revealed that, compared to baseline, the odds of choosing a plant-based menu increased significantly under both the order nudge (OR = 1.95, 95% CI [1.55, 2.45], *p* <.001) as well as under the order + recommendation nudge (OR = 1.95, 95% CI [1.56, 2.44], *p* <.001), representing increases of 4.7 and 4.4% points, respectively (Table 5). There was no significant difference in plant-based menu selection between the order nudge and the order + recommendation nudge (OR = 1.00, 95% CI [0. 80, 1.25], *p* =.992).

**Table 4:** Absolute numbers of chosen menus by phase of intervention

	Animal-based menus	Plant-based menus	Total menus
Baseline (Phase 1)	7,584	1,488	9,072
Order Nudge (Phase 2)	6,472	1,726	8,198
Order + Oral Recommendation Nudge (Phase 3)	7,668	2,011	9,679

**Table 5:** Main and subgroup analyses of sex and age on the effect of menu choice

	Baseline (Phase 1) (*N* = 2,215)		Order nudge (Phase 2) (*N* = 2,085)		Order + oral recommendation nudge (Phase 3) (*N* = 2,275)		Phase 1 vs. Phase 2			Phase 1 vs. Phase 3			Phase 2 vs. Phase 3		
	AB	PB	AB	PB	AB	PB	OR	95% CI	P*	OR	95% CI	P*	OR	95% CI	P*
Total sample (%)^a^	83.6	16.4	78.9	21.1	79.2	20.8	1.95	1.55–2.45	< 0.001	1.95	1.56–2.44	< 0.001	1.00	0.80–1.25	0.992
Female subgroup (%)^b^	81.4	18.6	76.6	23.4	77.3	22.7	2.01	1.48–2.72	< 0.001	1.84	1.37–2.48	< 0.001	0.92	0.69–1.23	0.560
Male subgroup (%)^b^	89.1	10.9	83.6	16.4	83.3	16.7	1.86	1.31–2.65	< 0.001	2.06	1.46–2.90	< 0.001	1.12	0.78–1.57	0.563
Aged 18–35 (%)^c^	68.3	31.7	69.2	30.8	66.9	33.1	1.37	0.62–3.01	< 0.426	1.02	0.46–2.25	< 0.969	0.74	0.33–1.63	0.458
Aged 36–64 (%)^c^	84.6	15.4	78.8	21.2	80.4	19.6	2.17	1.46–3.24	< 0.001	2.09	1.42–3.08	< 0.001	0.96	0.65–1.42	0.848
Aged ≥ 65 (%)^c^	87.2	12.8	82.5	17.5	81.7	18.3	1.96	1.45–2.64	< 0.001	2.13	1.60–2.84	< 0.001	1.09	0.82–1.44	0.560

AB: Animal-based menu; PB: Plant-based menu, N: Number of patients

* P-values were calculated using generalized linear mixed model

^a^ Generalized linear mixed model adjusted for age and sex

^b^ Generalized linear mixed model adjusted for age

^c^ Generalized linear mixed model adjusted for sex

### Subgroup analyses

Follow-up subgroup analyses adjusted for age and sex, respectively, revealed similar patterns. Among female patients, plant-based menu selection increased from 18.6% at baseline to 23.4% in the order nudge phase and 22.7% in the recommendation phase, representing increases of 4.8 and 4.1% points, respectively (Table 5). These increases were statistically significant, with the odds of choosing a plant-based menu twice as high under the order nudge (OR = 2.01, 95% CI [1.48, 2.72], *p* <.001) and nearly twice as high under the order + recommendation nudge (OR = 1.84, 95% CI [1.37, 2.46], *p* <.001). No significant difference was observed between the two intervention phases (OR = 0.92, 95% CI [0.69, 1.23], *p* =.560). Among male patients, plant-based choices increased from 10.9% at baseline to 16.4% under the order nudge and 16.7% under the recommendation nudge– an increase of 5.5 and 5.8% points, respectively. These effects were also statistically significant (order nudge: OR = 1.86, 95% CI [1.31, 2.65], *p* <.001; order + recommendation nudge: OR = 2.06, 95% CI [1.46, 2.90], *p* <.001), with no significant difference between the two nudges (OR = 1.12, 95% CI [0.78, 1.57], *p* =.563).

Among middle-aged adults (36–64 years), the proportion of choosing plant-based meals increased from 15.4% at baseline to 21.2% under the order nudge and 19.6% under the order + recommendation nudge– an absolute increase of 5.8 and 4.2% points, respectively (order nudge: OR = 2.17, 95% CI = [1.46–3.24], *p* <.001; order + recommendation: OR = 2.09, 95% CI = [1.42–3.08], *p* <.001). Older adults (aged ≥ 65) showed similar increases of 4.7 and 5.5% points, respectively, and were also nearly twice as likely to choose a plant-based meal under the respective nudge conditions (order nudge: OR = 1.96, 95% CI [1.45, 2.64], *p* <.001; order + recommendation nudge: OR = 2.13, 95% CI [1.60, 2.84], *p* <.001). In both age groups, the differences between nudge conditions were not significant (middle-aged: OR = 0.96, 95% CI [0.65, 1.42], *p* =.848; older adults: OR = 1.09, 95% CI [0.82, 1.44], *p* =.560). For younger adults (18–35 years), plant-based meal selection did not significantly differ across any of the three phases (all *p* >.001 (Table 5). Full estimates of all generalized linear mixed models are presented in Supplementary Table 1.

To test the robustness of our primary findings, we repeated the analyses with the excluded patients who were part of more than one intervention phase. In total, 11,104 menu choices from 903 patients were analyzed. Compared to the main sample, the subsample of excluded patients showed a smaller but similar effect: The probability of choosing a plant-based menu was increased in both intervention phases compared to baseline order nudge: OR = 1.68; order + recommendation nudge: OR = 1.73, both *p* <.001). Full results of all subgroup analyses are provided in Supplementary Table 2.

## Discussion

This study showed that a centrally implemented nudge– re-ordering an inpatient menu by placing the plant-based dish first– increased patients’ choices of plant-based lunch menus. The likelihood of selecting a plant-based dish nearly doubled during the order nudge phase compared to baseline (OR = 1.95, *p* <.001), corresponding to an absolute increase of 4.7% points in plant-based menu selection, suggesting that the order nudge was both statistically and practically effective in shifting dietary decisions. During the order nudge + recommendation nudge, the odds of selecting a plant-based dish remained high (OR = 1.95, *p* <.001), with a 4.4% point increase compared to baseline. Nudging further showed to be effective across demographic groups, with significant increases ranging between 4.2 and 5.8% points– most notably among male patients and adults aged 36–64. In total, the intervention led to 523 additionally ordered plant-based menus.

Our results align with studies demonstrating that rearranging menu options can effectively shift dietary choices towards healthier, more sustainable choices [27, 28, 29]. When an additional recommendation nudge was added, the effect of the order nudge was not exceeded (OR = 1.00, *p* =.992). In contrast to Venema et al. (2024) [37], our multicomponent nudge therefore had no greater effect than our single order nudge. As we did not test the recommendation nudge in isolation, we cannot disentangle whether the absence of an additional effect reflects inefficacy of the recommendation or is due to the combination of nudges which may potentially dilute individual effects. However, previous studies have shown positive effects of recommendation nudges [32, 33, 37], which suggests that implementation or design limitations, rather than ineffectiveness, may explain the lack of an added effect.

A possible explanation may be noncompliance of the menu assistants: although all assistants were previously trained in how to recommend the plant-based dishes to patients, a lack of intrinsic motivation to follow the instructions, time constraints and/or a busy clinical environment may have led them to skip the recommendation. Likely, the order nudge may have already reached the majority of patients susceptible to nudging, thereby overshadowing any additional influence of the recommendation. Alternatively, patients may have already made up their mind when receiving the printed menus upon arrival. The timing of the recommendation itself might have therefore limited its impact, reducing its persuasive power. Elsewise, previous research has focused on written recommendations [32, 33, 37]. In contrast, due to practical considerations, the present study employed an oral recommendation. Oral and written recommendations may differ in their effectiveness, which warrants further investigation. Moreover, research has shown that doctors are perceived as the most influential in guiding patients’ dietary decisions– more so than healthcare providers or even dietitians [38]. Recommendation nudges within healthcare may therefore be more impactful if framed as the “doctor’s recommendation” or, ideally, delivered by physicians themselves.

The significant increase of plant-based meals in both phase 2 and 3, however, suggests that the nudging intervention had a sustained impact across different groups and indicates that the effect was likely driven by the intervention rather than differences in participant characteristics. Although the absolute percentage point increases of 4.7 and 4.4 were modest, they represent meaningful shifts in dietary choices, given rather low baseline rates of 16.4% of choosing plant-based meals. Considering that the proportion of chosen plant-based meals remained below the 33% level– which might be expected if all three menu options were equally chosen– the near doubling of the odds of selecting a plant-based meal (OR = 1.95 in phase 2; OR = 1.95 in phase 3) further underscores the intervention’s effectiveness in shifting dietary behaviors. Notably, a similar pattern emerged in a subgroup analysis of participants who appeared in multiple phases (OR = 1.68 in phase 2; OR = 1.73 in phase 3), further supporting the robustness of the effect even under conditions where carry-over influences could be expected.

While the order nudge proved effective, some contextual factors may have limited the magnitude of its potential. For example, the plant-based dish was offered alongside two animal-based alternatives, possibly reducing the likelihood of selecting the plant-based meal - especially if it was perceived as less appealing in terms of taste or familiarity. Moreover, the digital ordering system (Ariadne) could not be modified to align with the redesigned paper menus, which placed the plant-based option first. Hence, the ordering system continued to present the animal-based dish first. If the menu assistants followed this sequence when presenting the menu options to patients, the plant-based dish may have not consistently been promoted in the order as intended, potentially limiting the overall impact of the order nudge.

Despite these contextual limitations, our analysis revealed interesting patterns related to participant demographics. Consistent with previous literature [17, 18, 19, 20], our initial analysis found that female sex predicted plant-based menu selection. However, subgroup analyses confirmed that nudging successfully influenced menu choices across both sexes, contrasting findings that women are more likely to be influenced by nudges towards sustainable food choices than men [39]. In fact, male participants showed larger absolute increases in plant-based meal selection (+ 5.8) during the order nudge, while women showed higher relative increases in their likelihood to choose plant-based meals (OR = 2.01) compared to males (OR = 1.84). Nudging was therefore effective across sexes regardless of initial dietary tendencies, and may both encourage plant-based meal choices among those with lower baseline preferences (e.g. men) and among those already more receptive (e.g. women).

Our analysis also confirmed that younger age predicted a higher likelihood of selecting plant-based meals [17, 18, 19, 20]. For instance, 31.7% of younger adults chose the plant-based menu at baseline compared to only 15.4% of middle-aged and 12.8% of older adults. However, the nudging interventions did not lead to an increase in plant-based meal selections, suggesting that nudging was less effective in the young age group. In contrast, middle-aged and older adults showed significant increases under both nudging interventions. The lack of an intervention effect among younger adults may suggest a ceiling effect, where those already inclined toward plant-based choices could not be further influenced by the nudge. Elsewise, younger individuals may have been less responsive to the applied nudging strategies compared to older adults. After all, while sex and age may both shape baseline preferences in plant-based dietary choices in the same direction, their moderating role on nudging effectiveness might differ.

### Strengths and limitations

A strength of this study is that intervention effects of participants who were in multiple phases showed a similar direction (OR = 1.68 in phase 2; OR = 1.73 in phase 3), suggesting that the observed effects were robust even under conditions where carry-over influences might have occurred. However, although the study shows potential for promoting healthy and climate-friendly food choices during hospitalization, it also features limitations. First, a quasi-experimental design without randomization and a control group limits the ability to infer causal implications. While data were obtained anonymously without active patient involvement, reducing the risk of detection bias, further research should be conducted in controlled designs.

Another key limitation concerns the implementation fidelity of the intervention. Limited resources did not allow us to monitor the adherence of the menu assistants to present the plant-based dish first and to recommend it explicitly. We therefore do not know to what extent the adherence of the assistants to the intervention protocol influenced our findings. Furthermore, the recommendation nudge was only tested in combination with the order nudge, which restricts our ability to calculate its individual contribution.

### Implications for future research and practice

Future study designs may include a control group, to better assess whether the observed effects are indeed due to the intervention or if they may be attributed to other factors. Moreover, increasing the temporal distance between the respective nudging intervention phases would reduce the likelihood of participant overlap across phases. To eliminate the necessity to rely on the menu assistants, recommendations may, for example, be implemented in the printed version of the weekly menu. However, it needs to be noted that, if the plant-based dish is consistently labeled as recommended while alternative options remain available, patients may question the authenticity or trustworthiness of the suggestion. Alternatively, educating the menu assistants about the benefits of a planetary diet might increase their intrinsic motivation to make recommendations. In the present study, they were only instructed to make the recommendations without explicitly mentioning the benefits.

Additionally, future research may focus solely on the effect of a single recommendation nudge, to isolate its specific contribution to dietary changes in hospital settings. Moreover, adopting other nudging methods such as making plant-based meals the default version and providing meat only upon request [40] would be interesting to investigate further.

Finally, if nudging temporarily influenced food choices, its long-term influence on patients’ dietary choices and habits remains uncertain. Patients may revert to familiar cooking and eating habits after their hospitalization [41]. Additional education or skills-based programs to encourage lasting changes towards healthy and sustainable eating might be needed. After all, follow-up studies may investigate long-lasting dietary changes of patients beyond hospitalization.

## Conclusion

This study investigated the effectiveness of different nudging strategies on choosing plant-based meals in adult hospital inpatients as part of a healthier and more sustainable diet. Our findings suggest that nudging can be a simply to implement, scalable and effective strategy towards healthier, more sustainable dietary choices in hospital inpatients, benefiting both the individual and the planet. Future research should investigate the differential effectiveness of different nudges, preferably in controlled settings.

## Electronic supplementary material

Below is the link to the electronic supplementary material.

Supplementary Material 1

## Data Availability

Availability of Data and Materials: The dataset analyzed during the study will be available from the corresponding author on reasonable request.

## Abbreviations

PHD: Planetary Health Diet
