# Supplementary material for: Exploring nudging strategies for plant-based dietary choices in hospital patients: a quasi-experimental study
Source: Int J Behav Nutr Phys Act. 2025 Jul 1;22:86. doi: 10.1186/s12966-025-01793-w (PMC12211476; doi:10.1186/s12966-025-01793-w)
Supplement: Supplementary file 1 — Supplementary Material 1 [file 12966_2025_1793_MOESM1_ESM.docx]

**Supplementary material to ‘****Exploring Nudging Strategies for Plant-based Dietary Choices in Hospital Patients: A Quasi-experimental Study’ by Kristin Hünninghaus, Hannah Caroline Schäfer, Maik Plonka, Rebeca Montejano Vallejo, Gustav Dobos, Heidemarie Haller**

**Supplementary Table 1.** Generalized linear mixed model estimates for the main and subgroup analysis (N = 6,575).

| **Model** | | **Estimate** | **Standard Error** | **p** |
| --- | --- | --- | --- | --- |
| **Total** | (Intercept) | -1.662 | 0.185 | <0.001 |
|  | Phase 2 | 0.666 | 0.117 | <0.001 |
|  | Phase 3 | 0.667 | 0.114 | <0.001 |
|  | Sex* | -0.723 | 0.094 | <0.001 |
|  | Age | -0.021 | 0.003 | <0.001 |
| **Female** | (Intercept) | -1.374 | 0.220 | <0.001 |
|  | Phase 2 | 0.697 | 0.154 | <0.001 |
|  | Phase 3 | 0.611 | 0.151 | <0.001 |
|  | Age | -0.024 | 0.003 | <0.001 |
| **Male** | (Intercept) | -2.946 | 0.337 | <0.001 |
|  | Phase 2 | 0.622 | 0.181 | <0.001 |
|  | Phase 3 | 0.724 | 0.175 | <0.001 |
|  | Age | -0.015 | 0.004 | <0.001 |
| **Aged 18–35** | (Intercept) | -1.947 | 0.375 | <0.001 |
|  | Phase 2 | 0.318 | 0.400 | 0.426 |
|  | Phase 3 | 0.016 | 0.406 | 0.969 |
|  | Sex* | -0.999 | 0.356 | 0.005 |
| **Aged 36–64** | (Intercept) | -3.217 | 0.248 | <0.001 |
|  | Phase 2 | 0.776 | 0.204 | <0.001 |
|  | Phase 3 | 0.738 | 0.198 | <0.001 |
|  | Sex* | -0.905 | 0.165 | <0.001 |
| **Aged 65+** | (Intercept) | -2.992 | 0.147 | <0.001 |
|  | Phase 2 | 0.672 | 0.152 | <0.001 |
|  | Phase 3 | 0.756 | 0.146 | <0.001 |
|  | Sex* | -0.484 | 0.120 | <0.001 |

*For variable sex, female was used as the reference category.

**Supplementary Table 2.** Subgroup analyses on the effect of menu choice in the sample of excluded patients (N = 903)

|  | **Baseline  (Phase 1)**  **(N = 627)** | | **Order nudge (Phase 2)**  **(N = 759)** | | **Order + oral recommendation nudge (Phase 3) (N = 590)** | | **Phase 1 vs. Phase 2** | | | **Phase 1 vs. Phase 3** | | | **Phase 2 vs. Phase 3** | | |
| --- | --- | --- | --- | --- | --- | --- | --- | --- | --- | --- | --- | --- | --- | --- | --- |
|  | AB | PB | AB | PB | AB | PB | OR | 95% CI | P* | OR | 95% CI | P* | OR | 95% CI | P* |
| Total sample (%)^a^ | 87.5 | 12.5 | 81.1 | 18.9 | 81.6 | 18.4 | 1.68 | 1.42 – 1.99 | <.001 | 1.73 | 1.42 – 2.10 | <.001 | 1.03 | 0.86 – 1.22 | .764 |
| Female subgroup (%)^b^ | 85.9 | 14.1 | 80.0 | 20.0 | 78.5 | 21.5 | 1.62 | 1.28 – 2.04 | <.001 | 1.90 | 1.44 – 2.49 | <.001 | 1.17 | 0.92 – 1.49 | .194 |
| Male subgroup (%)^b^ | 90.9 | 9.1 | 86.0 | 14.0 | 86.2 | 13.8 | 1.75 | 1.36 – 2.24 | <.001 | 1.56 | 1.19 – 2.05 | .001 | 0.89 | 0.70 – 1.14 | .360 |
| Aged 18-35 (%)^c^ | 72.1 | 27.9 | 66.0 | 34.0 | 75.3 | 24.7 | 0.65 | 0.33 – 1.29 | .220 | 0.58 | 0.30 – 1.11 | .100 | 0.88 | 0.45 – 1.75 | .728 |
| Aged 36-64 (%)^c^ | 87.7 | 12.3 | 81.9 | 18.1 | 79.9 | 20.1 | 1.65 | 1.29 – 2.10 | <.001 | 2.00 | 1.52 – 2.64 | <.001 | 1.21 | 0.95 – 1.56 | .120 |
| Aged ≥ 65 (%)^c^ | 91.2 | 8.8 | 83.5 | 16.5 | 84.9 | 15.1 | 1.99 | 1.53 – 2.58 | <.001 | 1.88 | 1.40 – 2.54 | <.001 | 0.94 | 0.74 – 1.21 | .655 |
| AB: Animal-based menu; PB: Plant-based menu, N: Number of patients *P-values were calculated using generalized linear mixed model ^a^ Generalized linear mixed model adjusted for age and sex  ^b^ Generalized linear mixed model adjusted for age  ^c^ Generalized linear mixed model adjusted for sex | | | | | | | | | | | | | | | |
